# Randomized Comparison of the Efficacies and Tolerabilities of Three Artemisinin-Based Combination Treatments for Children with Acute Plasmodium falciparum Malaria in the Democratic Republic of the Congo

**DOI:** 10.1128/AAC.02682-14

**Published:** 2014-09

**Authors:** M. A. Onyamboko, C. I. Fanello, K. Wongsaen, J. Tarning, P. Y. Cheah, K. A. Tshefu, A. M. Dondorp, F. Nosten, N. J. White, N. P. J. Day

**Affiliations:** a Mahidol-Oxford Tropical Medicine Research Unit, Faculty of Tropical Medicine, Mahidol University, Bangkok, Thailand; b Centre for Tropical Medicine, Nuffield Department of Medicine, University of Oxford, Oxford, United Kingdom; c Kinshasa School of Public Health, University of Kinshasa, Kinshasa, Democratic Republic of the Congo; d Shoklo Malaria Research Unit, Faculty of Tropical Medicine, Mahidol University, Bangkok, Thailand

## Abstract

An open-label, randomized controlled trial was carried out in 2011–2012 in the Democratic Republic of the Congo to test the efficacy, safety, and tolerability of the artemisinin-based combination treatments dihydroartemisinin-piperaquine, amodiaquine-artesunate, and artemether-lumefantrine. Six hundred eighty-four children aged 3 to 59 months with uncomplicated Plasmodium falciparum malaria were randomly allocated to each study arm. Children were hospitalized for 3 days, given supervised treatment, and followed up weekly for 42 days. All regimens were well tolerated and rapidly effective. The median parasitemia clearance half-life was 2.2 h, and half-lives were similar between arms (*P* = 0.19). The PCR-uncorrected cure rates by day 42 were 73.0% for amodiaquine-artesunate, 70.2% for artemether-lumefantrine, and 86.3% for dihydroartemisinin-piperaquine (*P* = 0.001). Early treatment failure occurred in three patients (0.5%), one in each arm. The PCR-corrected cure rates were 93.4% for amodiaquine-artesunate, 92.7% for artemether-lumefantrine, and 94.3% for dihydroartemisinin-piperaquine (*P* = 0.78). The last provided a longer posttreatment prophylactic effect than did the other two treatments. The day 7 plasma concentration of piperaquine was below 30 ng/ml in 47% of the children treated with dihydroartemisinin-piperaquine, and the day 7 lumefantrine concentration was below 280 ng/ml in 37.0% of children who received artemether-lumefantrine. Thus, although cure rates were all satisfactory, they could be improved by increasing the dose. (This study has been registered with the International Standard Randomized Controlled Trial Number Register [www.isrctn.org] under registration no. ISRCTN20984426.)

## INTRODUCTION

The Democratic Republic of the Congo (DRC) is one of the five countries with the greatest malaria burden in the world (1). The current national policy for the treatment of uncomplicated Plasmodium falciparum malaria consists of amodiaquine-artesunate (AA) or artemether-lumefantrine (AL), although artemether-lumefantrine, which was introduced in 2010, has very limited availability in the public sector. Amodiaquine-artesunate remains the most widely distributed antimalarial therapy in DRC. It was introduced in 2006, replacing sulfadoxine-pyrimethamine, which is now used only as an intermittent preventive treatment in pregnancy. The distribution and access of antimalarials in the rural areas of the country are organized through the public sector, whereas in the urban setting the private sector is predominant. Due to the civil unrest that has affected the country for many years, there is a paucity of data concerning the efficacy of antimalarial drugs in DRC. Available studies show substantial geographic variation in therapeutic efficacy, with similar variation in the prevalence of polymorphic alleles in P. falciparum genes associated with parasitological failure (2–4). This reflects the vast geographical area of the country.

Dihydroartemisinin-piperaquine (DP) is an artemisinin-based combination therapy (ACT) with a good safety and tolerability profile which is as effective as other ACTs in areas of endemicity in Asia and Africa (5). Piperaquine is a bisquinoline with a chemical structure similar to those of chloroquine and amodiaquine. The long terminal elimination half-life (∼23 days) provides lengthy posttreatment chemoprophylaxis, and the simple once-daily dosage regimen facilitates adherence (6). Dihydroartemisinin-piperaquine efficacy in Africa has so far been good, although no data are available for DRC.

The aim of this trial was to assess the efficacy of amodiaquine-artesunate for the treatment of uncomplicated P. falciparum malaria in children in Kinshasa, DRC, 5 years after its introduction as a first-line treatment, and to compare this with the efficacies of potential alternatives, dihydroartemisinin-piperaquine and artemether-lumefantrine, the latter recently added to the first-line treatment policy. The study was registered with the International Standard Randomized Controlled Trial Number Register (www.isrctn.org) under registration no. ISRCTN20984426.

## MATERIALS AND METHODS

### Study area.

The study was carried out in a research center located in an urban district of Kinshasa (DRC). Malaria transmission in the area is intense and perennial, with two annual peaks corresponding to the rainy seasons.

### Patient population.

Patients attending the health center with suspected clinical malaria were screened and enrolled in the study if they met the following inclusion criteria: age 3 to 59 months, weight of ≥5 kg, monoinfection with P. falciparum, parasitemia density between 2,000 and 200,000 asexual parasites/μl, axillary body temperature of ≥37.5°C or history of fever in the preceding 24 h, hemoglobin level of ≥5.0 g/dl, and ability to take oral medication. Patients with severe malaria (7), mixed-species malarial infection, any other significant concomitant illness, underlying disease, malnutrition, known allergy to any of the study drugs, or a clear history of adequate antimalarial treatment with drugs in the previous 72 h or who were taking prophylaxis with drugs having antimalarial activity were excluded. All cases excluded from the trial were referred to the hospital for diagnosis and treatment.

### Trial design.

This was an individually randomized, open-label study, comparing three fixed-dose oral artemisinin-based combination therapies: dihydroartemisinin-piperaquine (DP), artemether-lumefantrine (AL) and amodiaquine-artesunate (AA).

### Treatment.

Patients were randomly allocated to receive one of the three study treatments.

AA (artesunate-amodiaquine; Winthrop Sanofi, Kenya) was administered once a day for 3 days according to body weight at a mean dosage of 3.8 mg/kg of body weight/day of artesunate and 10.2 mg/kg/day of amodiaquine. Three types of fixed-dose tablets were used, containing artesunate at 25 mg, 50 mg, or 100 mg plus amodiaquine at 67.5 mg, 135 mg, or 270 mg. Tablets were administered according to the manufacturer's instructions: 4.5 to 8.9 kg of weight, 1 tablet of 25 mg artesunate/67.5 mg amodiaquine; 9 to 17.9 kg, 1 tablet of 50 mg artesunate/135 mg amodiaquine; and 18 to 35.9 kg, 2 tablets of 100 mg artesunate/270 mg amodiaquine.

DP (Dartepp; Guilin Pharmaceutical China; 40 mg dihydroartemisinin and 320 mg piperaquine, each tablet) was administered once a day for 3 days according to body weight with the following scheme: 5 to 7.9 kg, 0.5 tablet; 8 to 9.9 kg, 0.75 tablet; 10 to 14.9 kg, 1 tablet; 15 to 20.9 kg, 1.5 tablets; and 21 to 29.9 kg, 2.0 tablets. This dosage scheme was different from the one recommended by the manufacturer (5 to 10 kg, 0.5 tablet; 11 to 20 kg, 1 tablet; and 21 to 35 kg, 2.0 tablets). We used 5 intervals of weight instead of 3 to improve the therapeutic dose of dihydroartemisinin at the upper limit of the range for each interval. The mean dosage was 3.3 mg/kg/day of dihydroartemisinin and 26.6 mg/kg/day of piperaquine.

AL (Coartem; Novartis, Switzerland) was administered in 6 doses over 3 days (0, 8, 24, 36, 48, and 60 h). Each tablet contained 20 mg artemether and 120 mg lumefantrine, and the mean dose was 2.0 mg/kg of artemether and 12.7 mg/kg of lumefantrine for each dose. Tablets were administered according to the manufacturer's instructions: 5 to 14.9 kg, 1 tablet; 15 to 24.9 kg, 2 tablets; 25 to 34.9 kg, 3 tablets; and >35 kg, 4 tablets.

Tablets were administered under medical supervision and with 100 ml of milk (the fat in the milk improves drug absorption). Patients were observed for 1 h after drug ingestion. The full dose was repeated if the patient vomited within 30 min, and a half-dose was given if the patient vomited within 1 h.

Patients who failed treatment were treated with intravenous (i.v.) quinine if severe (20-mg salt/kg of body weight loading dose followed by 10 mg/kg every 8 h) or oral quinine if uncomplicated (10 mg/kg three times daily for 7 days) according to national policy. Parenteral artesunate was not available.

All children were hospitalized for 3 days and followed up actively once a week for 42 days after treatment. Caretakers were invited to come back to the center or to contact the study nurse in the case that the child was unwell. If the patient did not report for the scheduled visits, every effort was made to locate him or her at the home address. At each visit, the medical history, clinical signs and symptoms, body temperature, and a blood sample for parasitemia were collected.

### Sample size.

For the calculation of the sample size, we assumed a cure rate of 95% with AL, 99% with DP, and 85% with AA. A sample size of 621 patients would have been adequate to detect a 10% difference between the standard treatment (AA) and AL or DP at the 5% level and with 90% power. The sample size was increased by 10% to allow for loss to follow-up (final sample size, *n* = 684).

### Randomization, sequence generation, type, allocation concealment mechanism, and implementation.

The randomization sequence, in blocks of 15, was computer generated and numerically sequenced. Opaque envelopes containing the study drug name were prepared at the Mahidol Oxford Tropical Medicine Research Unit (MORU), Bangkok, Thailand. Patients were enrolled by the study physician and assigned to treatment by the study nurse who opened the next consecutively numbered envelope. Once an envelope was opened, the patient was considered included in the study.

### Outcome measurements.

The primary outcome measure was the PCR-corrected cure rate by day 42. Secondary outcome measures were parasite and fever clearance and occurrence of adverse events (AE). Treatment outcome was established according to the standard WHO classification (8). Early treatment failure (ETF) was defined as (i) danger signs or severe malaria on day 1, 2, or 3, in the presence of parasitemia; (ii) parasitemia on day 2 higher than that on day 0, irrespective of axillary temperature; (iii) parasitemia on day 3 with axillary temperature of ≥37.5°C; and (iv) parasitemia on day 3 of ≥25% of count on day 0. Late clinical failure (LCF) was defined as (i) danger signs or severe malaria in the presence of parasitemia on any day between day 4 and day 42 in patients who did not previously meet any of the criteria of early treatment failure and (ii) axillary temperature of ≥37.5°C in the presence of parasitemia on any day between day 4 and day 42 in patients who did not previously meet any of the criteria of early treatment failure. Late parasitological failure (LPF) was defined as the presence of parasitemia between day 7 and day 42 with a temperature of <37.5°C in patients who did not previously meet any of the criteria of early treatment failure or late clinical failure. Adequate clinical and parasitological response (ACPR) was defined as absence of parasitemia on day 42, irrespective of axillary temperature, in patients who did not previously meet any of the criteria of early treatment failure, late clinical failure, or late parasitological failure.

Safety reporting was performed according to the ICH Harmonized Tripartite Guideline for Good Clinical Practice (9).

### Laboratory methods.

Asexual and sexual malaria parasites were identified and counted on Giemsa-stained thick films and reported per 200 leukocytes (WBC), assuming a total WBC count of 8,000/μl (10). Slides were declared negative after examination of at least 100 high-power microscopy fields. Parasite species was determined on the thin film. The laboratory technicians were blinded to the treatment received by individual patients. The blood film prepared during the screening was considered the admission slide.

Blood films were prepared at baseline and 6 and 12 h and then repeated every 12 h until 2 consecutive negative blood films were observed. Parasite clearance was assessed (i) as the time for the parasite count to decrease to 50% of its initial value (PC_50_) and (ii) as parasite clearance rate derived from the log-linear section of the log parasitemia-time curve and expressed as the parasite clearance half-life (PCt_1/2_; log_e_2/parasite clearance rate).

To compare the PCt_1/2_ measured in this study with the more recent data collected in 2013 during the Tracking Resistance to Artemisinin Collaboration (TRAC) project, all slides were read a second time after the study was terminated by the same microscopist team using a different counting technique: if more than 20 parasites were seen on the thick smear after 10 fields, parasitemia per 1,000 erythrocytes (RBC) was counted on the thin smear. Below that threshold, parasites were counted on the thick smear per 500 WBC.

Hemoglobin was measured on admission using a portable photometer (HemoCue Hb201+; Angelholm, Sweden). Thereafter, the hematocrit was measured at baseline, daily during the hospitalization, and at days 7 and 14 of the follow-up by microhematocrit centrifugation (Hawksley Haematospin 1400; Hawksley & Sons, Ltd., United Kingdom).

Total and differential WBC counts were assessed daily during the hospitalization and at day 7 and 14 of the follow-up (Sysmex automated hematology analyzer).

Liver function tests were performed, and aspartate aminotransferase (AST), alanine aminotransferase (ALT), and creatinine levels were measured from plasma at the hospital laboratories (SEAC-Screenmaster) at baseline and 48 h.

A dried blood spot (DBS) was prepared at admission, daily during the hospitalization, and at each follow-up visit for further molecular analysis.

### Drug analysis.

A random sample of tablets of DP was analyzed for content and quality at the Department of Pharmacology of MORU, and 2 ml of venous blood was taken at day 7 from 246 consecutive patients to measure plasma concentrations of lumefantrine and piperaquine.

### Molecular analysis.

Paired filter paper samples from enrollment and the follow-up day on which parasites were detected by microscopy were analyzed at the Shoklo Malaria Research Unit (SMRU) to distinguish between recrudescence and reinfection. Parasite DNA was purified (QiaAmp DNA microkit; Qiagen, United Kingdom), and the three polymorphic markers MSP-1, MSP-2, and GLURP were genotyped. A recrudescent infection was defined as one that matched in size at least one allele of each marker between the first and second samples. If any pair of alleles of a polyclonal primary infection was detected during a second episode, this was considered a recrudescence.

### Ethical approval.

The study was approved by the Oxford University Research Ethic Committee (OXTREC), the Institutional Review Board of Kinshasa School of Public Health (KSPH), and the Ministry of Public Health of DRC. A verbal consent was obtained from caretakers before screening children for malaria and anemia. A written consent form was obtained from caretakers whose children fulfilled all inclusion criteria before enrolling the patient in the study.

The study was monitored regularly by a qualified internal monitor (MORU Clinical Trials Support Group) for adherence to Good Clinical Practice (GCP) regulations. All Investigators and the Research Ethic Committee (REC) of KSPH were notified of serious adverse events (SAEs).

### Statistical analysis.

Data were double entered in Microsoft Access 2007 and validated using Epi Info 6.4b (CDC, Atlanta, GA, USA). Statistical analyses were performed using STATA v.11 (StataCorp LP, College Station, TX). Descriptive statistics were used to summarize demographic data and baseline values. For the per-protocol analysis, χ^2^ was used to compare proportions. Analysis of variance (ANOVA) was used for normally distributed continuous data, and the nonparametric Kruskal-Wallis test was used to analyze continuous data with a nonnormal distribution.

For the intention-to-treat (ITT) analysis, the log rank test was used to test the equality of the survivor function across groups and Cox regression was used to estimate hazard ratio of infections posttreatment.

The overall fractional reduction in hematocrit was defined as the difference between the patient's lowest level of hematocrit and that at baseline (i.e., pretreatment) divided by the hematocrit at baseline. The percentages of patients whose hematocrit fell >20% or 25% were compared between groups. No interim analyses for efficacy or futility were done.

## RESULTS

### ITT analysis and deviations from study protocol.

**FIG 1:**
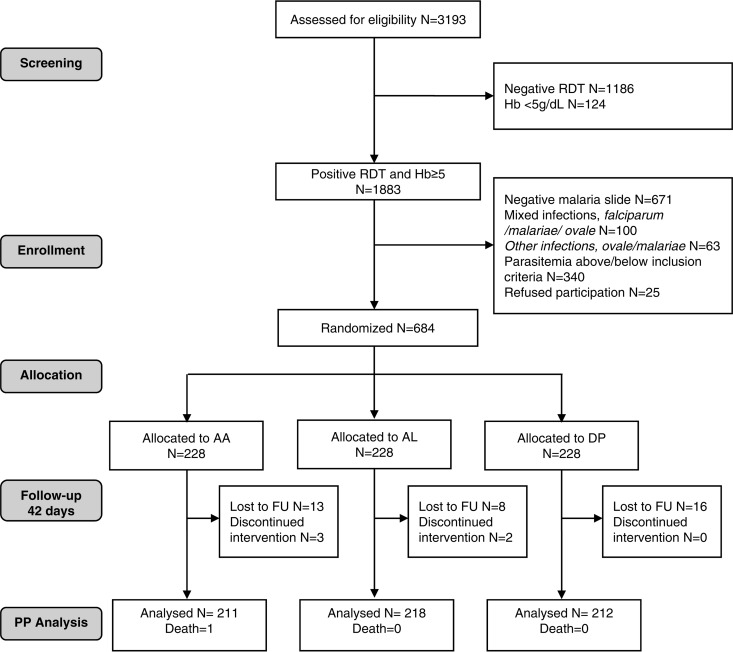
CONSORT flow chart. FU, follow-up; RDT, rapid diagnostic test; Hb, hemoglobin; PP, per protocol.

Between September 2011 and November 2012, 684 patients were included in the study, 228 in each treatment group. Forty-two patients (6.1%) discontinued the study: 5 children were withdrawn during the hospitalization because the families changed their minds and 37 were lost to follow-up between days 7 and 42 (DP, 16; AL, 10; AA, 16). One patient, in the AA group, died at day 29 from causes unrelated to malaria or the study drug. These cases were not included in the per-protocol analysis, and they were censored on the last day that the patients were visited by the doctor and tested for malaria in the intention-to-treat (ITT) analysis. The flow of patients through the study is outlined in the patient flow diagram (Fig. 1).

### Baseline characteristics and treatment.

At enrollment, patients had similar demographic, clinical, and parasitological characteristics (Table 1). The tablets of DP contained an average of 35.6 mg dihydroartemisinin (89%) and 306 mg piperaquine (95.5%). This was compared to the Eurartesim (Sigma Tau) product, which contained an average of 40.7 mg dihydroartemisinin (102%) and 300 mg piperaquine (94%).

**TABLE 1:** Baseline characteristics of children at enrollment by treatment group

Characteristic	Value by treatment group:		
	AA	AL	DP
No. of patients at admission	228	228	228
Female/male ratio	115/113	105/123	105/123
Mean age in mo (range)	35.3 (3–59)	33.5 (5–59)	33.7 (5–59)
Mean wt in kg (95% CI)	12.5 (12.1–12.8)	12.5 (12.1–12.9)	12.3 (11.9–12.7)
Axillary temp (°C), median (range)	37.5 (36.0–40.5)	37.2 (36.0–40.8)	37.1 (36.0–40.2)
Splenomegaly, no. positive/total no. (%)	72/226 (31.9)	84/228 (36.8)	88/228 (38.6)
Hepatomegaly, no. positive/total no. (%)	3/228 (1.32)	1/228 (0.44)	0/228
No. of P. falciparum parasites*/*μl			
Median (range)	30,066 (2,093–199,840)	30,119 (2,040–199,720)	35,207 (2,126–199,960)
Geometric mean (95% CI)	25,179 (21,188–29,921)	25,681 (21,828–30,216)	30,403 (25,657–36,026)
Patients with >150,000 parasites/μl, no. positive/total no. (%)	21/228 (9.2)	14/228 (6.1)	29/228 (12.7)
Mean hemoglobin, g/dl (95% CI)	9.7 (9.4–9.9)	9.7 (9.5–10.0)	9.6 (9.4–9.8)

### Drug efficacy. (i) Per-protocol analysis.

The cure rates by day 42 (primary outcome), PCR uncorrected, were similar in patients treated with AA (73.5%) and those treated with AL (70.6%), whereas the cure rate was significantly higher in patients treated with DP (86.8%) (*P* = 0.001) (Table 2. In the follow-up period, 145 children were diagnosed with a second episode of malaria (starting as early as day 16); most of these cases were new infections, and only 30 (21%) were confirmed by PCR as recurrent infections. Among the new infections, there were 12 cases of Plasmodium malariae and 1 of Plasmodium ovale. For 9 patients, the PCR was unsuccessful; as we could not ascertain if these 9 cases were new or recurrent infections, we excluded them from the PCR-corrected analysis. After correcting the results for the new infections, the cure rates were comparable in the three groups: 93.4% for AA (95% confidence interval [CI], 89.1% to 96.3%), 92.7% for AL (95% CI, 88.4% to 95.7%), and 94.3% for DP (95% CI, 90.3% to 97.0%) (*P* = 0.76).

**TABLE 2:** Per-protocol analysis: efficacy by treatment at day 42

Outcome	Value by treatment group:			*P* value
	AA	AL	DP	
Total no. allocated to treatment	228	228	228	
No. withdrawn or lost to follow-up by day 42	16	10	16	
No. of deaths	1	0	0	
No. of evaluable patients	211	218	212	
Results, PCR uncorrected, no. (%)				
Early treatment failure	1 (0.47)	1 (0.46)	1 (0.47)	
Late clinical failure	17 (8.1)	12 (5.5)	10 (4.7)	
Late parasitological failure	39 (18.5)	52 (23.9)	18 (8.5)	
Adequate clinical and parasitological response	154 (73.0)	153 (70.2)	183 (86.3)	0.001
PCR results on recurrent episodes, no.				
New infections with P. falciparum	41	39	16	
New infections with P. malariae/P. ovale	2	10	1	
Recrudescences of P. falciparum	10	11	9	
Undetermined PCR result^*a*^	3	4	2	
Results, PCR corrected: adequate clinical and parasitological response, no. (%)	197 (93.4)	202 (92.7)	200 (94.3)	0.78

a The samples were collected, but the PCR results were undetermined; cases were excluded from the PCR-corrected analysis.

Early treatment failure occurred in three patients (0.5%), one in each arm. Data are reported also using day 28 cure rates as the endpoint (Table 3).

**TABLE 3:** Per-protocol analysis: efficacy by treatment at day 28

Outcome	Value by treatment group:			*P* value
	AA	AL	DP	
Follow-up not completed by day 28, no.	12	5	10	
Results, PCR uncorrected: adequate clinical and parasitological response, no. (%)	183 (86.7)	190 (87.1)	206 (97.2)	0.001
Results, PCR corrected: adequate clinical and parasitological response, no. (%)	207 (98.1)	211 (96.8)	208 (98.1)	0.578

### (ii) Intention-to-treat analysis.

**FIG 2:**
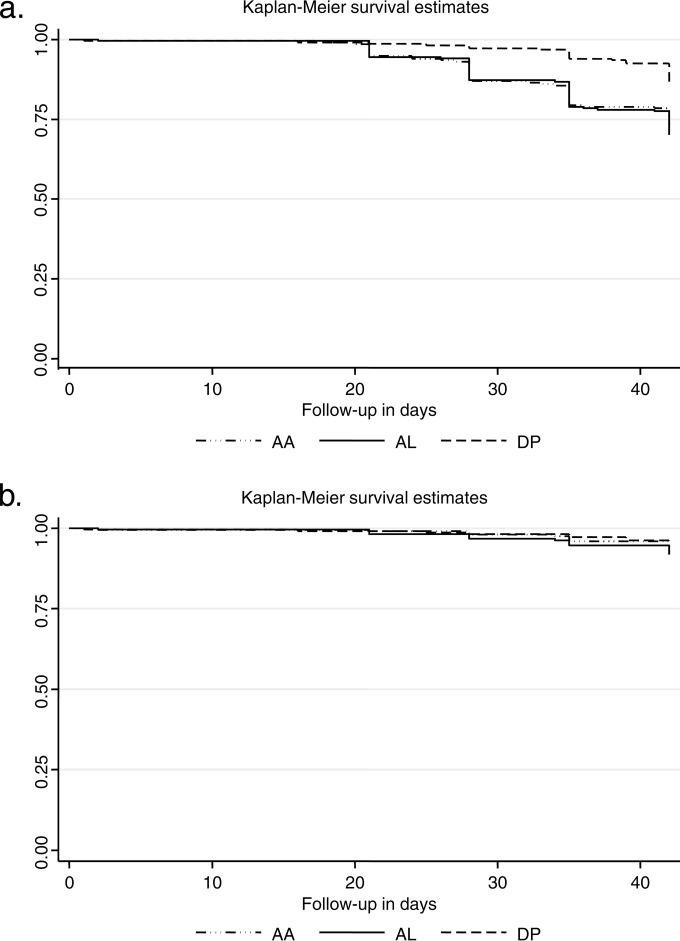
Intention-to-treat analysis: Kaplan-Meier plots of failure rates (*y* axis) without (a) and with (b) PCR correction.

The ITT analysis showed similar results (log rank test for equality of survivor functions, PCR uncorrected, χ^2^ = 18.83, *P* = 0.0001, and PCR corrected, χ^2^ = 1.00, *P* = 0.61) (Fig. 2). The risk (hazard ratio) of having a second episode of malaria (either new or recurrent) in the follow-up period was 1.5 times higher in the AA arm and 2.4 higher in the AL arm than in the DP arm (*P* > 0.0001). The results were not affected by age or initial parasitemia.

### Fever clearance.

On admission, 26.8% of patients had fever (axillary temperature of ≥37.5°C). For the other patients, the parent or guardian reported a history of fever in the preceding 24 h as the main reason for seeking a doctor at the health center. After 24 h, 97% of children were afebrile and there was a significantly higher proportion of children with fever in the AL group at day 2 (*P* = 0.003; Table 4).

**TABLE 4:** Fever clearance: percentage still febrile on day 0 to day 3 by treatment group

Time in days	No. positive/total no. (%) by treatment group:			*P* value
	AA	AL	DP	
0	63/228 (27.6)	65/228 (28.5)	55/228 (24.1)	0.53
1	3/226 (1.3)	11/226 (4.9)	8/228 (3.5)	0.1
2	1/226 (0.4)	9/226 (4.0)	1/228 (0.4)	0.003
3	2/226 (0.9)	3/226 (1.3)	5/227 (2.2)	0.50

### Parasitemia clearance.

**FIG 3:**
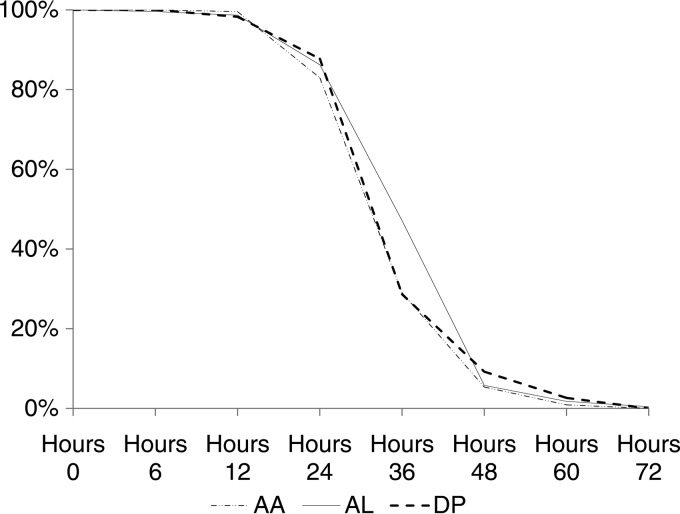
Parasite positivity rate (*y* axis) by day (*x* axis) and treatment group.

All treatments were associated with a rapid clearance of parasitemia. The parasite positivity rate (proportion of children with a positive slide at day 2) was significantly higher in the AL arm (*P* < 0.001; Fig. 3). Accordingly, the median PC_50_ was significantly longer for AL (8.4 h; range, 0.2 to 23.9 h; *n* = 214) than for AA (5.7 h; range, 0.1 to 24.3 h; *n* = 204) and DP (6.5 h; range, 0.1 to 34.4 h; *n* = 212) (*P* < 0.001) (Table 5). The median PCt_1/2_ was 2.2 h (range, 1.0 to 6.3 h; *n* = 657) with no significant differences between arms, indicating similar efficacies of the three different artemisinin derivatives (*P* = 0.08) (Table 6).

**TABLE 5:** Time (hours) to clear 50% of parasitemia by treatment

Treatment group	No. of observations used for estimation	Time (h) to clear 50% of parasitemia		
		Median	Range	IQR^*a*^
AA	204	5.71	0.09–24.26	5.39
AL	214	8.44	0.18–23.85	5.64
DP	212	6.54	0.08–34.40	5.12
Total	630	7.31	0.08–34.40	5.63

a IQR, interquartile range.

**TABLE 6:** Slope half-life (based on the slope of the log-linear portion of the parasite clearance curve)

Treatment group	No. of observations used for estimation	Slope half-life (h)		
		Median	Range	IQR^*a*^
AA	214	2.15	1.05–4.35	0.87
AL	223	2.23	1.05–6.32	0.73
DP	220	2.13	0.97–4.85	0.76
Total	657	2.18	0.97–6.32	0.82

a IQR, interquartile range.

### Gametocytemia.

**FIG 4:**
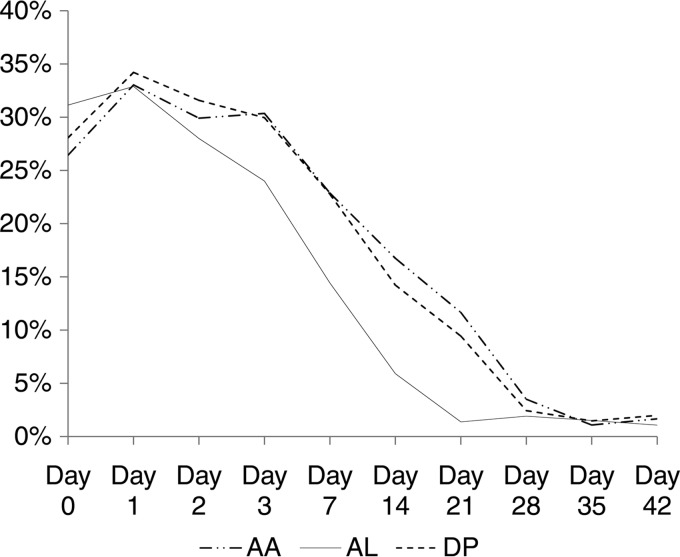
Gametocyte positivity rate (*y* axis) by treatment days 0 to 42 (*x* axis).

On admission, 28.5% (*n* = 195/684) of patients were gametocytemic with no significant differences between groups. Treatment with AL resulted in lower gametocyte carriage rates than did the other two treatments in the follow-up period, days 7 to 21 (*P* < 0.001) (Fig. 4). In 7 children, gametocytemia was microscopically detectable from admission until day 35 (AA = 4, DP = 2, and AL = 1), and in 2 children treated with DP, it was detectable until day 42. In a number of children, gametocytes were not detected on admission blood smears but became apparent in the first 72 h of treatment with no significant differences between arms. New appearance of gametocytes was, however, uncommon from day 7 onward.

### Hematology.

The mean packed cell volume (PCV) at admission was 30.1% (95% CI, 29.8% to 30.5%; range, 13% to 42%). In the first week, there was an overall mean fractional reduction in the PCV of 10% (standard deviation [SD], 8.3) with no differences between arms (*P* = 0.65) (Table 7). Hyperparasitemic children (≥150,000/μl) were those most affected, with a mean fractional reduction of 15.1% (95% CI, 7.4 to 8.7) compared to 8.1% (95% CI, 13.8 to 16.3) in those who were not hyperparasitemic (*P* < 0.001).

**TABLE 7:** Mean fractional reductions in PCV and numbers of patients whose reductions in PCV were >20% or >25% compared to the value at admission by treatment group

Treatment group	Day 0–3			Day 0–7			Day 0–14		
	Mean % (SD)	% (no. positive/total no.) of patients with PCV reduction:		Mean % (SD)	% (no. positive/total no.) of patients with PCV reduction:		Mean % (SD)	% (no. positive/total no.) of patients with PCV reduction:	
		>20%	>25%		>20%	>25%		>20%	>25%
AA	10.0 (8.2)	12.4 (28/226)	6.2 (14/226)	10.3 (8.2)	12.8 (29/226)	6.6 (15/226)	10.4 (8.2)	12.8 (29/226)	6.6 (15/226)
AL	9.0 (8.4)	11.8 (27/228)	4.4 (10/228)	9.4 (8.4)	11.8 (27/228)	4.4 (10/228)	9.7 (8.7)	12.7 (29/228)	4.8 (11/228)
DP	9.3 (7.9)	9.7 (22/228)	5.7 (13/228)	10.0 (8.2)	12.7 (29/199)	6.1 (14/228)	10.2 (8.2)	13.2 (30/228)	6.6 (15/228)
Total	9.4 (8.1)	11.3 (77/682)	5.4 (37/682)	9.9 (8.3)	12.5 (85/682)	5.7 (39/682)	10.1 (8.3)	12.9 (88/682)	6.0 (41/682)
*P* value	0.41	0.62	0.68	0.51	0.94	0.55	0.65	0.99	0.65

A reduction of >25% of the initial PCV value was observed in 14.9% of hyperparasitemic children and 2.3% of nonhyperparasitemic children (*P* < 0.001).

Ten patients developed decompensated anemia within 4 days of recruitment and required a blood transfusion: 3 in the AA group, 4 in the AL group, and 3 in the DP group (*P* = 0.62). The risk of receiving a blood transfusion was 6.5 times higher in the hyperparasitemic children (95% CI, 2.90 to 22.3; *P* = 0.005). By day 14, the levels were comparable to those at admission in all patients.

The median WBC counts were similar between the treatment groups on recruitment and at each day of follow-up with an increase from day 0 to day 7 to normal values (Table 8). Neutrophil counts decreased gradually from baseline values until day 14 with no differences between groups on any day. Mild neutropenia (<1,000 neutrophils/μl) was observed in 2.5% of patients at enrollment, and between days 1 and 7, the neutrophil count fell below 1,000/μl in 18% of patients (123/684) with no differences between groups (*n* = 40 in AA, 40 in AL, and 43 in DP; *P* = 0.92). Fourteen of these patients developed severe neutropenia (<500 neutrophils/μl) (2, 4, and 8 in the AA, AL, and DP groups, respectively; *P* = 0.13).

**TABLE 8:** Median WBC and differential counts and interquartile ranges at days 0, 7, and 14 by treatment group

Treatment group and day	*n*	Count μl^−1^					
		WBC		Neutrophil		Lymphocyte	
		Median	IQR^*a*^	Median	IQR	Median	IQR
AA							
0	227	6,800	4,000	3,096	2,311	2,919	2,170
7	216	8,200	4,475	2,677	1,846	4,651	2,884
14	212	7,000	3,075	2,014	1,288	4,489	2,234
AL							
0	228	6,650	4,050	2,829	2,414	2,841	2,388
7	221	7,600	4,000	2,496	1,706	4,623	2,817
14	217	6,900	2,900	2,118	1,306	4,300	2,240
DP							
0	228	6,875	5,050	3,201	3,254	3,075	2,730
7	219	7,600	3,750	2,520	1,745	4,550	2,394
14	215	7,700	3,700	2,352	1,476	4,590	2,792

a IQR, interquartile range.

### Hepatotoxicity.

Mean serum levels of aspartate aminotransferase (U/liter), alanine aminotransferase (U/liter), and creatinine (mg/dl) were similar at baseline among groups. With minor fluctuations (a trend toward a reduction for AST and ALT), the mean levels at day 2 remained similar to those of day 1 with no statistical differences between groups (data not shown).

### Tolerability.

During the first day, children treated with DP vomited within 1 h of the first dose significantly more often (*n* = 21; 9.2%) than did children treated with AA (*n* = 10; 4.4%) or AL (*n* = 5; 2.2%) (*P* = 0.03). The second dose of AL—administered after 8 h—was vomited in seven cases, and taking this into account, the overall difference between treatments during the first day was not significant (*P* = 0.06). These children were all given a second dose of the drug. On the second day, there was no difference in vomiting postdose between children treated with AA (15; 6.6%) and those treated with DP (17; 7.5%), whereas in the AL arm only 2 (0.9%) cases vomited after the first dose and none after the second dose (*P* = 0.02).

### Adverse events.

At least one adverse event was reported in 37.4% of patients during the posttreatment period that was not present on admission or increased in intensity and was classified as possibly or probably related to the study drug. Most AEs were graded as of minor or moderate intensity. The most frequent AEs were weakness, anorexia, and gastrointestinal disorders (nausea, vomiting, abdominal pain, and diarrhea). However, these symptoms overlap known malaria symptomatology. Anorexia and weakness were reported more frequently in children treated with AA than in those treated with DP and AL during the second and third days of treatment (day 1, 15.2% [34/224] for AA, 5.3% [12/227] for AL, and 5.8% [13/228] for DP [*P* = 0.0001], and day 2, 8.2% [17/207] for AA, 2.3% [5/220] for AL, and 4.0% [9/224] for DP [*P* = 0.013]). There were otherwise no differences in the numbers of AEs between treatment groups. In 17 cases, the adverse event was graded as severe or life-threatening. All cases were classified as unlikely to be related to the study treatment. Ten patients developed decompensated anemia during the hospitalization and required a blood transfusion (described above). There was one case of severe skin eruption 18 days posttreatment (DP), one case of chickenpox 12 days posttreatment (AL), 1 case of abscess 11 days posttreatment (DP), 1 case of leukocytosis 2 weeks posttreatment (AL), and 3 cases of asthenia (2 AL and 1 AA). One child in the AA group died in a different hospital at day 29. The cause of death was unknown, but the death was considered unlikely to have been caused by either malaria or the drug treatment.

### Plasma lumefantrine levels at day 7.

One hundred twenty-one samples of venous blood were collected at day 7 from patients who received AL (Table 9). The median concentration of lumefantrine in the blood was 377 ng/ml (range, 57.1 to 1,150 ng/ml). Drug levels were positively correlated with body weight (*r* = 0.22; test for trend, *P* = 0.005). The plasma level was significantly lower in children weighing <15 kg (median, 309 ng/ml; range, 57.1 to 1,080; *n* = 87) than in those weighing ≥15 kg (median, 473 ng/ml; range, 108 to 1,150; *n* = 34; *P* = 0.01). Accordingly, 43.7% of children weighing <15 kg had a plasma level of ≤280 ng/ml, considered the cutoff for therapeutic efficacy (11), compared to 20.6% in those weighing ≥15 kg (*P* = 0.018). The 7 children in this subsample with a PCR-confirmed recrudescence had a median level of 429 ng/ml (range, 147 to 703 ng/ml), not significantly different from those who successfully cleared the infection (376 mg/ml; range, 57.1 to 1,150; *P* = 0.8; *n* = 114).

**TABLE 9:** Lumefantrine plasma level at day 7

Body wt (kg)	No. analyzed	Median (range) dose received, mg/kg	Median day 7 LM^*a*^ level, ng/ml (range)	% of samples with ≤280 ng/ml
5–9.9	22	27.9 (24.5–45.3)	294.5 (63.4–1,050)	45.5
10–14.9	65	20.0 (17.1–24.0)	364.0 (57.1–1,080)	43.1
15–20.9	34	30.0 (24.0–32.0)	473 (108–1,150)	20.6
Total	121	24.0 (17.1–45.3)	421.9 (57.1–1,150)	37.2

a LM, lumefantrine.

### Plasma piperaquine levels at day 7.

One hundred twenty-five samples were collected from venous blood at day 7 from patients who received DP (Table 10). The median concentration of piperaquine was 31.4 ng/ml (range, 10.9 to 189.0), and drug levels were positively correlated with body weight (*r* = 0.22; test for trend, *P* = 0.04). In 47.2% (59/125) of patients, the plasma level was below 30 ng/ml, the previously published threshold associated with therapeutic efficacy (12), and the 3 patients with a PCR-confirmed recrudescence had piperaquine levels of 16.3, 31.4, and 33.1 ng/ml, respectively.

**TABLE 10:** Piperaquine plasma levels at day 7

Body wt (kg)	No. analyzed	Median (range) dose received, mg/kg	Median day 7 PQ^*a*^ level, ng/ml (range)	% of samples with ≤30 ng/ml
5–7.9	8	20.8 (20.3–33.3)	23.6 (10.9–65.2)	75
8–9.9	17	28.2 (17.2–30.0)	31.8 (16–70.8)	41.2
10–14.9	65	26.7 (22.1–32.0)	28.1 (12.6–135)	52.3
15–20.9	35	30.0 (26.7.0–32.0)	44.3 (15.8–189)	34.3
Total	125	28.2 (17.2–33.3)	31.4 (10.9–189)	47.2

a PQ, piperaquine.

## DISCUSSION

The efficacies of the three combination therapies tested in this trial were similar. The proportion of children with an adequate clinical and parasitological response was 93% for AL and 94% for DP, both rarely used in the area. These cure rates are similar to that of AA (93%), which has been extensively used in DRC since its introduction in 2006.

The PCR-corrected day 28 cure rate for AL in the present study was 96.8%, which is comparable to the 97.9% day 28 cure rate observed in the same area 3 years previously (13).

In the subsample analyzed, the drug level of piperaquine at day 7, reflecting the concentration of drug to which residual parasites are exposed and thus predictive of outcome, was suboptimal in 47% of patients. This confirms previous results (6, 14) showing that as small children have a higher body-weight-normalized oral clearance, they need a higher dose than the one currently recommended. Of the children who received artemether-lumefantrine, 37% had a suboptimal day 7 lumefantrine level, with the smaller children having the lowest levels. These results are comparable to those observed in Uganda (15). As there was no evidence of delayed parasitemia clearance suggestive of artemisinin resistance, the PCR-confirmed treatment failures observed in the current study are likely caused by either low drug exposure, as suggested by the pharmacokinetic (PK) results, or parasite resistance to the nonartemisinin partner drug. The former is much more likely. Moreover, in high-transmission areas, the chances that the recurrent infection contains a parasite with the same genotype as that in the primary infection are higher than those in low-transmission areas. This, along with the persistence of gametocytes in the blood, can lead in some cases to a misclassification of recurrent infections as recrudescences (16).

In this clinical trial, we measured efficacy of treatments administered under supervision, with a glass of milk, and retreatment was given if the first dose was vomited. Drug exposure, in a nontrial setting (generally unsupervised), is expected to be lower (15). Dose optimization and schedule changes are a priority for the ACT, especially in small children, to ensure adequate drug exposure (14, 17).

Although the efficacies in terms of ACPR rates of the three ACTs were comparable, children treated with DP were at lower risk of having a second episode of malaria during the follow-up period because of the longer posttreatment prophylactic effect of DP related to the longer plasma half-life of piperaquine. This chemoprophylactic effect is important in areas of endemicity such as the study area and makes this drug a good candidate for replacing sulfadoxine pyrimethamine for intermittent preventive treatment in pregnant women and children (18, 19).

This study population was characterized by hyperparasitemia, and the initial high levels of parasitemia affected, as expected, the recovery of hematocrit after the initial episode of malaria, but not the treatment efficacy. The initial level of gametocytemia was also high (30%), and AL was significantly more effective in clearing the sexual stages than were the other ACTs. Data in literature on the gametocytocidal properties of the different ACT are conflicting, and the effect, if any, on malaria transmission is unclear (20).

The median PCt_1/2_ was 2.2 h (range, 1.0 to 6.3 h; *n* = 657) and comparable to the results observed in 2013 during the TRAC project, 2.2 h (range from 1.2 to 4.6 h; *n* = 60) (21). The difference that we observed between PC_50_s with the three therapies (but not with the PCt_1/2_) could be attributed to the relatively slow conversion of artemether to dihydroartemisinin (artemether half-life of 2.0 h) compared to artesunate (artesunate half-life of 0.84 h) and dihydroartemisinin in the acute phase of malaria (22), resulting in a significantly longer lag phase (the initial flat part of the parasite clearance profile) for AL and/or the lower dosage of artemether (20). The PC_50_ includes the lag phase of the parasite clearance curve, whereas the PCt_1/2_ is based on the log-linear phase alone.

The three combinations were well tolerated, and there were no significant differences in the types and numbers of adverse events between arms.

### Conclusions.

The three combinations tested were equally efficacious and well tolerated for the treatment of children with acute uncomplicated P. falciparum malaria. Dihydroartemisinin-piperaquine had the longest-lasting chemoprophylactic effect which prevented repeated clinical attacks in the treated children. The recommended dosage of DP provides suboptimal piperaquine plasma concentrations, particularly in small children.

## References

[1] World Health Organization. 2013 World malaria report: 2013. World Health Organization, Geneva, Switzerland

[2] Alker AP Kazadi WM Kutelemeni AK Bloland PB Tshefu AK Meshnick SR 2008 Dhfr and dhps genotype and sulfadoxine-pyrimethamine treatment failure in children with falciparum malaria in the Democratic Republic of Congo. Trop. Med. Int. Health 13:1384–1391. 10.1111/j.1365-3156.2008.02150.x 19055622 PMC2765712

[3] Bonnet M Broek I van Herp M Urrutia PP van Overmeir C Kyomuhendo J Ndosimao CN Ashley E Guthmann JP 2009 Varying efficacy of artesunate+amodiaquine and artesunate+sulphadoxine-pyrimethamine for the treatment of uncomplicated falciparum malaria in the Democratic Republic of Congo: a report of two in-vivo studies. Malar. J. 8:192. 10.1186/1475-2875-8-192 19664280 PMC2734861

[4] Mobula L Lilley B Tshefu A Rosenthal P 2009 Short report: resistance-mediating polymorphisms in Plasmodium falciparum infections in Kinshasa, Democratic Republic of the Congo. Am. J. Trop. Med. Hyg. 80:555–558 19346374

[5] Naing C Mak JW Aung K Wong JY 2013 Efficacy and safety of dihydroartemisinin-piperaquine for treatment of uncomplicated Plasmodium falciparum malaria in endemic countries: meta-analysis of randomised controlled studies. Trans. R. Soc. Trop. Med. Hyg. 107:65–73. 10.1093/trstmh/trs019 23222952

[6] Tarning J Zongo I Some FA Rouamba N Parikh S Rosenthal PJ Hanpithakpong W Jongrak N Day NP White NJ Nosten F Ouedraogo JB Lindegardh N 2012 Population pharmacokinetics and pharmacodynamics of piperaquine in children with uncomplicated falciparum malaria. Clin. Pharmacol. Ther. 91:497–505. 10.1038/clpt.2011.254 22258469 PMC3736305

[7] WHO. 2013 Management of severe malaria: a practical handbook, 3rd ed. WHO, Geneva, Switzerland

[8] WHO. 2009 Methods for surveillance of antimalarial drug efficacy. WHO, Geneva, Switzerland

[9] ICH-GCP. 1996 ICH Harmonised Tripartite Guideline for Good Clinical Practice E6(R1). International Conference on Harmonisation of Technical Requirements for Registration of Pharmaceuticals for Human Use, Geneva, Switzerland

[10] WHO. 2010 Basic malaria microscopy, 2nd ed. WHO, Geneva, Switzerland

[11] Ezzet F Mull R Karbwang J 1998 Population pharmacokinetics and therapeutic response of CGP 56697 (artemether + benflumetol) in malaria patients. Br. J. Clin. Pharmacol. 46:553–561 9862244 10.1046/j.1365-2125.1998.00830.x PMC1873796

[12] Price RN Hasugian AR Ratcliff A Siswantoro H Purba HL Kenangalem E Lindegardh N Penttinen P Laihad F Ebsworth EP Anstey NM Tjitra E 2007 Clinical and pharmacological determinants of the therapeutic response to dihydroartemisinin-piperaquine for drug-resistant malaria. Antimicrob. Agents Chemother. 51:4090–4097. 10.1128/AAC.00486-07 17846129 PMC2151469

[13] Tshefu AK Gaye O Kayentao K Thompson R Bhatt KM Sesay SS Bustos DG Tjitra E Bedu-Addo G Borghini-Fuhrer I Duparc S Shin CS Fleckenstein L 2010 Efficacy and safety of a fixed-dose oral combination of pyronaridine-artesunate compared with artemether-lumefantrine in children and adults with uncomplicated Plasmodium falciparum malaria: a randomised non-inferiority trial. Lancet 375:1457–1467. 10.1016/S0140-6736(10)60322-4 20417857

[14] WorldWide Antimalarial Resistance Network (WWARN) DP Study Group. 2013 The effect of dosing regimens on the antimalarial efficacy of dihydroartemisinin-piperaquine: a pooled analysis of individual patient data. PLoS Med. 10:e1001564. 10.1371/journal.pmed.1001564 24311989 PMC3848996

[15] Checchi F Piola P Fogg C Bajunirwe F Biraro S Grandesso F Ruzagira E Babigumira J Kigozi I Kiguli J Kyomuhendo J Ferradini L Taylor WR Guthmann JP 2006 Supervised versus unsupervised antimalarial treatment with six-dose artemether-lumefantrine: pharmacokinetic and dosage-related findings from a clinical trial in Uganda. Malar. J. 5:59. 10.1186/1475-2875-5-59 16854236 PMC1543643

[16] Greenhouse B Dokomajilar C Hubbard A Rosenthal PJ Dorsey G 2007 Impact of transmission intensity on the accuracy of genotyping to distinguish recrudescence from new infection in antimalarial clinical trials. Antimicrob. Agents Chemother. 51:3096–3103. 10.1128/AAC.00159-07 17591848 PMC2043236

[17] Staehli Hodel EM Guidi M Zanolari B Mercier T Duong S Kabanywanyi AM Ariey F Buclin T Beck HP Decosterd LA Olliaro P Genton B Csajka C 2013 Population pharmacokinetics of mefloquine, piperaquine and artemether-lumefantrine in Cambodian and Tanzanian malaria patients. Malar. J. 12:235. 10.1186/1475-2875-12-235 23841950 PMC3720542

[18] Nankabirwa JI Wandera B Amuge P Kiwanuka N Dorsey G Rosenthal PJ Brooker SJ Staedke SG Kamya MR 2014 Impact of intermittent preventive treatment with dihydroartemisinin-piperaquine on malaria in Ugandan schoolchildren: a randomized, placebo-controlled trial. Clin. Infect. Dis. 58:1404–1412. 10.1093/cid/ciu150 24621953 PMC4001293

[19] Lwin KM Phyo AP Tarning J Hanpithakpong W Ashley EA Lee SJ Cheah P Singhasivanon P White NJ Lindegardh N Nosten F 2012 Randomized, double-blind, placebo-controlled trial of monthly versus bimonthly dihydroartemisinin-piperaquine chemoprevention in adults at high risk of malaria. Antimicrob. Agents Chemother. 56:1571–1577. 10.1128/AAC.05877-11 22252804 PMC3294930

[20] Price RN 2013 Potential of artemisinin-based combination therapies to block malaria transmission. J. Infect. Dis. 207:1627–1629. 10.1093/infdis/jit079 23468055 PMC4627501

[21] Ashley E The spread of artemisinin resistance in falciparum malaria. N. Engl. Med. J., in press 10.1056/NEJMoa1314981 PMC4143591 25075834

[22] Suputtamongkol Y Newton PN Angus B Teja-Isavadharm P Keeratithakul D Rasameesoraj M Pukrittayakamee S White NJ 2001 A comparison of oral artesunate and artemether antimalarial bioactivities in acute falciparum malaria. Br. J. Clin. Pharmacol. 52:655–661. 10.1046/j.1365-2125.2001.01458.x 11736876 PMC2014567

